# Studies on the moss flora of the Bío-Bío Region of Chile: Part 3

**DOI:** 10.3897/phytokeys.77.10926

**Published:** 2017-02-03

**Authors:** Robert R. Ireland, Gilda Bellolio, Juan Larraín, Roberto Rodríguez

**Affiliations:** 1 Department of Botany, Smithsonian Institution, National Museum of Natural History, MRC-166, P.O. Box 37012, Washington, D.C., 20013-7012, U.S.A.; contact address: 2055 Carling Ave., Apt. 614, Ottawa, Ontario, K2A 1G6, Canada; 2 The Flora of Chile Project, Departamento de Botánica, Facultad de Ciencias Naturales y Oceanográficas, Universidad de Concepción, Casilla 160-C, Concepción, Chile; 3 Instituto de Biología, Pontificia Universidad Católica de Valparaíso, Campus Curauma, Av. Universidad 330, Curauma, Valparaíso, Chile

**Keywords:** Bryophyta, floristics, Bío-Bío Region, checklist, South America

## Abstract

This is the final report on the moss flora of the Bío-Bío Region (Región VIII) in south-central Chile where collections were made in 2001–2003. Reported in this paper are one species new to South America, four species new to Chile and 16 species new to the Region. With these new additions the total number of taxa in the Bío-Bío Region is 343, corresponding to 331 species and 12 infraspecific taxa. A complete checklist of the mosses for all the provinces in the Region is presented.

## Introduction

This is the final paper on the moss flora of the Bío-Bío Region of Chile, reporting the identifications for the specimens collected by R.R. Ireland and Gilda Bellolio in 2001–2003. Two earlier papers by Ireland et al. (2006, 2010) reported many mosses new for the Region, as well as for all of Chile. In the present paper are reports for one species new for South America, four new for Chile and 16 new for the Bío-Bío Region. All collection numbers are those of the two authors and the identifications were made by R.R. Ireland and Juan Larraín, with the exception of some problematic specimens that were identified by various other bryologists.

The original checklist of the mosses by He (1998) reported 190 taxa for the Region which was later updated by Müller (2009a, b) who reported 315 taxa. This number was subsequently increased to 323 taxa by Ireland et al. (2010). The total number of taxa now known for the Bío-Bío Region is 343, which is the current number reported in the present paper.

**Figure 1.**
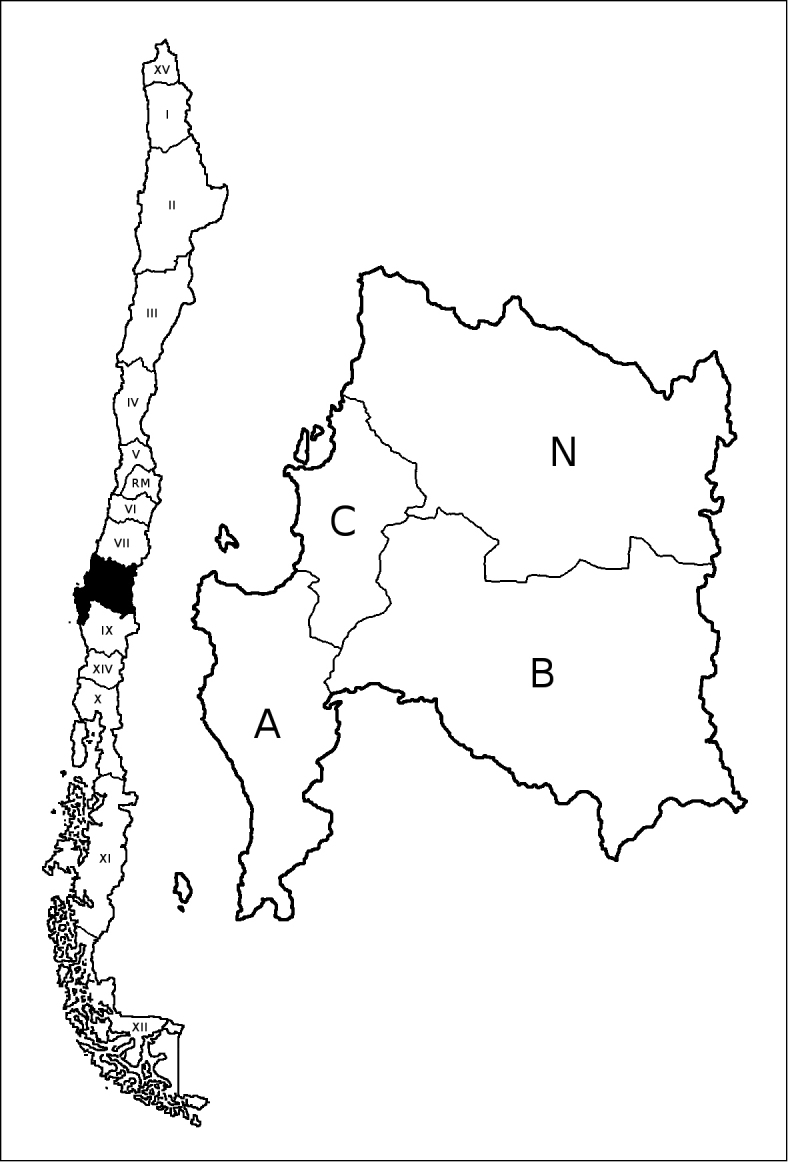
**Left.** Map of Chile showing in black the location of Bío-Bío Region (VIII) and the rest of Chilean Regions in Roman numerals (RM=Metropolitan Region). **Right.** Detail of Bío-Bío Region map showing the four provinces (A=Arauco, B=Bío-Bío, C=Concepción, N=Ñuble).

Since the publication of the first paper of this series on the Bío-Bío moss flora (Ireland et al. 2006), the administrative division of Chile has slightly changed, modifying the map given in the latter reference. In October 2007, two new regions were created after the breakup of the former I and X Regions: Region XV (Arica y Parinacota) became the northernmost region after splitting of the former I Region, whereas Region XIV (Los Ríos) was created to separate the administration of Valdivian area from the rest of the former Los Lagos Region (X Region, see Fig. 1).

## Methods

Phytogeographic information, climate and geomorphology for the four provinces (Arauco, Bío-Bío, Concepción, Ñuble) in the Region are all reported in the first paper of this project by Ireland et al. (2006). Voucher specimens of most of the collections are at CONC, MO and US, with many at NY. All numbers listed below correspond to specimens collected by Robert R. Ireland and G. Bellolio, unless otherwise indicated.

For the taxonomy we follow Müller (2009a), with the exception of the genus *Bryum* for which we followed the segregates outlined in Spence (2014). Segregates of *Racomitrium* are not recognized as suggested by Larraín et al. (2013). Synonyms are indicated for taxa treated under a different name in Müller (2009a) or on the Internet at www.tropicos.org.

For the biogeographical analysis, we grouped the taxa into seven groups: “Endemic” meaning taxa distributed in Chile, adjacent Argentina, sometimes extending into the Falkland Islands, South Georgia or the Juan Fernández Islands; “Wide Distribution” meaning taxa distributed in several continents or without a clear geographical pattern; “Southern Hemisphere” refers to widely distributed subantarctic species sometimes reaching tropical areas in the Andes or in South East Asia; “Bipolar” meaning taxa distributed in the temperate areas of both hemispheres; “Neotropical” meaning taxa widely distributed in Latin America and the Caribbean, including some species restricted to South America; “Gondwanic” referring to taxa distributed in southern South America and New Zealand and Australia; and “Neotropical + African” meaning taxa distributed both in the tropical and/or subtropical areas of both Latin America and Africa.

## Results

### Moss new to South America and Chile

***Sematophyllum harpidioides*** (Renauld & Cardot) F.D.Bowers – Arauco Prov., Sta. Aurora farm (Mininco), 38°01'S, 73°16'W. alt. ca. 486 m, *33513* (Det. B.H. Allen 2014); road from Curanilahue to Trongol, 19 km SE of Curanilahue, 37°34'S, 73°12'W, alt. ca. 990 m, *33095*, *33109*; Mocha Island, trail in National Park, 38°20'S, 75°53'W, alt. ca. 15–350 m, *33124*; Lincuyin, W of Lanalhue Lake, 37°57'S, 73°19'W, alt. ca. 250 m, *33464*, *33465*; Hwy. P-80-R, 13 km E of Antiquina, 38°03'S, 73°16'W, alt. 531 m, *33552*; 4 km W of Mahuique, 38°10'S, 73°14'W, alt. ca. 590 m, *33596*; road from Contulmo to Purén, 2 km S of Contulmo, 38°03'S, 73°13'W, alt. ca. 410 m, *33640*, *33648*.

Known only from Central America (Costa Rica and Honduras) according to B.H. Allen (MO) and specimens cited in Tropicos.

### Mosses new to Chile

***Bryum insolitum*** Cardot – Ñuble Prov., road to garbage dump, 6 km E of Quirihue, 36°17'S, 72°28'W, alt. ca. 570 m, *32347a* (det. by J.R. Spence 2016).

Known only from Mexico and Bolivia (Ochi 1980).

***Ptychostomum bimum*** (Schreber) J.R.Spence – Concepción Prov., waterfalls at toll booth near Sta. Juana, 1 km S of Curali, 37°15'S, 72°57'W, alt. ca. 135 m, *33934* (det. J.R. Spence 2014).

A widely distributed taxon (Spence 2014) that has been often lumped together with *Ptychostomum creberrimum* or with *Ptychostomum pseudotriquetrum* (Hedw.) G.Gaertn., B.Mey. & Scherb. (Zolotov 2000). Not previously reported for Chile.

***Ptychostomum creberrimum*** (Taylor) J.R.Spence & H.P.Ramsey – Bío-Bío Prov., Jaujau Farm (Mininco), 38°02'S, 71°57'W, alt. ca. 760 m, *35465* (det. J.R. Spence 2014).

A widely distributed taxon (Spence 2014) not previously reported for Chile.

***Ptychostomum inclinatum*** (Sw. ex Brid.) J.R.Spence – Ñuble Prov., Termas de Chillán & environs, 36°55'S, 71°42'W, alt. ca. 1660 m, *30585*, *30617* (both det. by J.R. Spence 2014).

A widely distributed taxon (Spence 2014) not previously reported for Chile.

### Mosses new to Bío-Bío Region of Chile

***Barbula costesii*** Thér. – Ñuble Prov., road from Quirihue to Cobquecura, 36°13'S, 72°36'W, alt. ca. 360 m, *32300*; road to Trapiche, 6 km NW of San Carlos, 36°21'S, 71°58'W, alt. ca. 290 m, *34772* (mixed with *Didymodon fuscus* (Müll.Hal.) J.A.Jiménez & Cano; both specimens det. by J.A. Jiménez 2010).

Reported from Regions V and XII (Müller 2009a).

***Bryoerythrophyllum berthoanus*** (Thér.) J.A.Jiménez – Ñuble Prov., Río Diguillín, Los Mañios, ca. 10 km SE of Recinto, 36°53'S, 71°38'W, alt. ca. 500 m, *30759* (mixed with *Didymodon fuscus* (Müll.Hal.) J.A.Jiménez & Cano; det. by J.A. Jiménez 2010).

Reported from Regions V, RM & VII (Müller 2009a). This record represents a southern extension of the distribution of this taxon.

***Bryoerythrophyllum campylocarpum*** (Müll.Hal.) H.A.Crum – Concepción Prov., Nonguén Valley, CORFO farm, 36°53'S, 72°59'W, alt. ca. 102 m, *31326* (det. by J.A. Jiménez 2010).

Reported for Region IX & Juan Fernández Is. (Müller 2009a). This record represents a northern extension of the continental Chile distribution of this taxon.

***Bryum longidens*** Thér. – Arauco Prov., Lanalhue Lake, 6 km S of Hwy. P-70, 37°55'S, 73°20'W, alt. ca. 33 m, *33447*; Hullinco Falls, 37°45'S, 73°22'W, alt. ca. 130 m, *33662*. Bío-Bío Prov., road from Tumeco to Florida, 2 km N from Hwy. 0–50, 36°57'S, 72°40'W, alt. ca. 190 m, *32053*. Concepción Prov., road from Santa Juana to La Laja, 19 km SE of Santa Juana, 37°16'S, 72°47'W, alt. ca. 135 m, *32879*; Ñuble Prov., road to garbage dump, 12 km E of Quirihue, 36°17'S, 72°25'W, alt. ca. 350 m, *32352* (all det. by J.R. Spence 2014–2016).

Previously known only from the type from Valparaíso Region (V). The name appeared after its synonymization under *Bryum pseudocapillare* Besch. made by Ochi (1980). This record represents a southern extension of the distribution of this taxon.

***Bryum pauperculum*** E.B.Bartram – Bío-Bío Prov., National Park Lake Laja, Los Barros Military Base, 37°27'S, 71°19'W, alt. ca. 1500 m, *34115*. Ñuble Prov., Shangri-La, old German refuge, 36°52'S, 71°31'W, alt. ca. 1350 m, *34462* (both det. by J.R. Spence 2014).

Previously known only from the type from Tierra del Fuego (Region XII). The name appeared after its synonymization under *Bryum pallens* Sw. made by Ochi (1982). These records represent a northern extension of the distribution of this taxon.

***Bryum puconense*** Herzog & Thér. – Arauco Prov., Mocha Island, 38°21'S, 73°56'W, alt. ca. 25 m, *33182*. Bío-Bío Prov., Saltillo del Itata, small falls on Itata River, 37°04'S, 72°09'W, alt. ca. 210 m, *34940* (both det. by J.R. Spence 2014).

Reported for Regions IX (Ochi 1982), and XI (Larraín 2016).

***Bryum zeballosicum*** Cardot & Broth. – Concepción Prov., road from Santa Juana to La Laja, 1 km E of Santa Juana, 37°10'S, 72°55'W, alt. ca. 50 m, *32852* (det. by J.R. Spence 2016).

Reported for Metropolitan Region (Müller 2009a). This record represents a southern extension of the distribution of this taxon.

***Calliergonella cuspidata*** (Hedw.) Loeske – Arauco Prov., Road from Curanilahue to Trongol, 19 km SE of Curanilahue, 37°34'S, 73°12'W, alt. 990 m, *33096*. Bío-Bío Prov., 37.7 km S of El Barco Lake, 38°02'S, 71°21'W, alt. ca. 950 m, *34249*. Concepción Prov., Universidad de Concepción campus, behind Facultad de Ciencias Naturales y Oceanográficas building, 36°49'S, 73°02'W, alt. ca. 30 m, *Larraín 32760* (det. by J. Larraín 2010).

Reported for Regions IX, X and XIV (Müller 2009a). This record represents a northern extension of the distribution of this taxon in Chile.

***Campylopus acuminatus*** Mitt. – Arauco Prov., Road from Curanilahue to Trongol, 19 km SE of Curanilahue, 37°34'S, 73°12'W, alt. 990 m, *33092*.

Reported for Regions IX – XII & XIV (Müller 2009a).

***Dendrocryphaea gorveana*** (Mont.) Paris & Schimp. – Concepción Prov., Reserva Nacional Nonguén, by trail next to Nonguén river, 36°53'S, 72°59'W, alt. ca. 300 m, *Larraín 32750* (det. by J. Larraín 2016).

Reported for Regions VII, and XIV-XI (Müller 2009a).

***Pohlia wilsonii*** (Mitt.) Ochyra – Concepción Prov., Patahual, 37°00'S, 72°59'W, alt. ca. 56.8 m, *30408*; Lonco & Villuco, 36°52'S, 73°00'W, alt. ca. 15 m, *31025*; Quebrada Honda, 36°41'S, 72°58'W, alt. ca. 104 m, *31689* (det. J. Larraín 2008); road to Las Pataguas, 3 km N from Hwy. 148, 36°49'S, 72°53'W, alt. ca. 222 m, *31761*, *31764*; road from Hualqui to Quilacoya, 5 km S of Hualqui, 37°01'S, 72°58'W, alt. ca. 107 m, *32081*; road from Dichato to Burca, 3 km N of Dichato, 36°31'S, 72°54'W, alt. ca. 89 m, *32219*. Ñuble Prov., road from La Achira to Trehuaco, 36°15'S, 72°45'W, alt. ca. 410 m, *32519*; Colmuvao, 36°18'S, 72°49'W, alt. 19 m, *32778*.

Reported for Regions II‒VI, RM, & XII (Müller 2009a).

***Rosulabryum campylothecium*** (Taylor) J.R.Spence – Concepción Prov., Caleta Chome, Punta Hualpén, 36°46'S, 73°12'W, alt. ca. 36 m, *31983*. Ñuble Prov., Cayumanque hill, 36°42'S, 72°31'W, alt. ca. 483 to 792 m, *31550* (both det. by J.R. Spence 2014).

Reported for Regions IV, V & IX (as *Bryum campylothecium* Taylor by Müller 2009a).

***Rosulabryum torquescens*** (Bruch & Schimp.) J.R.Spence – Concepción Prov., Park “Jorge Alessandri” (Compañia Manufacturera de Papeles y Cartones), 36°56'S, 73°09'W, alt. ca. 200-490 m, *32819* (det. by J.R. Spence 2014).

Reported for Regions VII & X (as *Bryum torquescens* Bruch ex DeNot by Müller 2009a).

***Rosulabryum viridescens*** (Welw. & Duby) Ochyra – Ñuble Prov., Las Trancas, 36°54'S, 71°30'W, alt. ca. 1300 m, *35912* (det. by J.R. Spence 2014).

Reported for Region IV & South Chile (as *Bryum viridescens* Welw. & Duby by Müller 2009a).

***Syntrichia costesii*** (Thér.) R.H.Zander – Ñuble Prov., Los Cipreses Farm, 36°56'S, 71°33'W, alt. ca. 1010 m, *35838*, *35842*; Las Trancas, 36°54'S, 71°30'W, alt. ca. 1300 m, *35893*.

Reported from Regions V, VII, IX – XII (Müller 2009a, Larraín 2016).

***Syntrichia socialis*** (Dusén) R.H.Zander – Bío-Bío Prov., Las Perlas Lake, 15 km NW of Cabrero, 36°57'S, 72°26'W, alt. ca. 300 m, *35954* (det. by R.H. Zander – reported in Tropicos).

Reported from Regions X & XII (Müller 2009a). This record represents a northern extension of the distribution of this taxon.

### Checklist of the mosses of the Bío-Bío Region of Chile

+++ New to South America; ++ New to Chile; + New to Bío-Bío Region (Región VIII); * Excluded from Chile; ** Excluded from Bío-Bío Region. (Provinces of the Bío-Bío Region represented by the following letters: A=Arauco; B=Bío-Bío; C=Concepción; N=Ñuble). All new provincial records since Ireland et al. (2010) paper, list the province in bold print indicating the corresponding voucher(s) in parenthesis.

***Acaulon uleanum*** Müll.Hal. – C

***Achrophyllum anomalum*** (Schwägr.) H.Rob. – B

***Achrophyllum magellanicum*** (Besch.) Matteri – A, B, C, N

var. ***oligodontum*** (Matteri) Matteri – B, C

***Acrocladium auriculatum*** (Mont.) Mitt. – A, B, N

***Amblystegium serpens*** (Hedw.) Schimp. – C, N

***Amphidium tortuosum*** (Hornsch.) Cufod. – A, B, C, N

***Ancistrodes genuflexa*** (Müll.Hal.) Crosby – A, B, C, N

***Andreaea acutifolia*** Hook.f. & Wilson – B

***Andreaea alpina*** Hedw. – N

***Andreaea rupestris*** Hedw. – A, B, N

***Andreaea subulata*** Harv. – B, N

***Anomobryum julaceum*** (Schrad. ex G.Gaertn., B.Mey. & Scherb.) Schimp. – A, B, C, N

***Aongstroemia gayana*** (Mont.) Müll.Hal. – B, C, N

***Arbusculohypopterygium arbuscula*** (Brid.) M.Stech, T.Pfeiff. & W.Frey – A, B

***Atractylocarpus patagonicus*** Herzog & Thér. – **B (35459)**, C

***Barbula convoluta*** Hedw. – C

+***Barbula costesii*** Thér. – **N (32300, 34772)**

***Bartramia bellolioella*** B.H.Allen & Ireland – A, N

***Bartramia ithyphylla*** subsp. ***patens*** (Brid.) Fransén – N

***Bartramia ithyphylloides*** Schimp. ex Müll.Hal. – A, B, C, N

***Bartramia mossmaniana*** Müll.Hal. – A, B, N

*Bartramia potosica Mont. – N (misidentified specimens that are all Bartramia ithyphylla subsp. patens –see Ireland et al. 2010, p. 43).

***Bartramia stricta*** Brid. – A, B, C, N

***Blindia magellanica*** Schimp. – A, B, C, N

***Brachymenium acuminatum*** Harv. – A, B, C, N

***Brachymenium exile*** (Dozy & Molk.) Bosch & Sande Lac. – C

***Brachymenium gilliesii*** (Hook.) A.Jaeger – B, N (Syn. *Bryum gilliesii* Hook.)

***Brachymenium meyenianum*** (Hampe) A.Jaeger – N

***Brachymenium robertii*** Broth. – C

***Brachytheciastrum microcollinum*** (E.B.Bartram) Ignatov & Huttunen – N

***Brachytheciastrum paradoxum*** (Hook.f. & Wilson) Ignatov & Huttunen – A, B, N

***Brachythecium albicans*** (Hedw.) Schimp. – N

***Brachythecium conostomum*** (Taylor) A.Jaeger – A, B, N

***Brachythecium rutabulum*** (Hedw.) Schimp. – A, B, N

***Brachythecium subpilosum*** (Hook.f. & Wilson) A.Jaeger – A, B, C, N

***Brachythecium subplicatum*** (Hampe) A.Jaeger – A, B, C, N

***Breutelia dumosa*** Mitt. – B, C

***Breutelia integrifolia*** (Taylor) A.Jaeger – B, N

***Breutelia subplicata*** Broth. – A, B, C, N

***Breutelia tomentosa*** (Sw. ex Brid.) A.Jaeger – C

+***Bryoerthrophyllum berthoanus*** (Thér.) J.A.Jiménez – **N (30759)** Mixed with *Didymodon fuscus* and filed in herbarium collections under this species.

+***Bryoerthrophyllum campylocarpum*** (Müll.Hal.) H.A.Crum – **C (31326)**

***Bryum argenteum*** Hedw. – A, B, C, N

***Bryum densifolium*** Brid. – N

***Bryum elegantulum*** Lorentz – C

++***Bryum insolitum*** Cardot – **N (32347a)**

+***Bryum longidens*** Thér. – **A (33447, 33662)**, **B (32053, 34815, 35759)**, **C (32879)**, **N (32352)**

***Bryum mucronatum*** Mitt. – N

***Bryum nivale*** Müll.Hal. – B

***Bryum orbiculatifolium*** Cardot & Broth. – B

+***Bryum pauperculum*** E.B.Bartram – **B (34115)**, **N (34462)**

***Bryum platyphyllum*** (Schwägr.) Müll.Hal. – **A (33251)**, B

+***Bryum puconense*** Herzog & Thér. – **A (33182)**, **B** (**34940**)

***Bryum revolutum*** Müll.Hal. – C

***Bryum subgracillimum*** Thér. – C

+***Bryum zeballosicum*** Cardot & Broth. – **C (32852)**

+***Calliergonella cuspidata*** (Hedw.) Loeske – **A (33096), B (34249), C (32760** Coll. *Larraín*)

***Calyptopogon mnioides*** (Schwägr.) Broth. – A, B, C, N

***Camptodontium cryptodon*** (Mont.) Reimers – A, B, N

***Campylopodium medium*** (Duby) Giese & J.-P.Frahm – A

+***Campylopus acuminatus*** Mitt. – **A (33092)**

***Campylopus aureonitens*** subsp. ***recurvifolius*** (Dusén) J.-P.Frahm – B, C

***Campylopus clavatus*** (R.Br.) Wilson – A, C

***Campylopus fragilis*** (Brid.) Bruch & Schimp. – A

***Campylopus incrassatus*** Müll.Hal. – A, B. C, N

***Campylopus introflexus*** (Hedw.) Brid. – A, B, C, N

***Campylopus laxoventralis*** Herzog ex J.-P.Frahm – C

***Campylopus modestus*** Cardot – C, N

***Campylopus pilifer*** Brid. – A, B, C, **N (30573, 30753, 30832, 32334, 32581, 34445, 34684, 35828, 36117, 36143)**

***Campylopus purpureocaulis*** Dusén – C

***Campylopus pyriformis*** (Schultz) Brid. – **A (32954, 33204, 36255)**, C, **N (32387)**

***Campylopus vesticaulis*** Mitt. – A, C, N

***Campylostelium saxicola*** (F.Weber & D.Mohr) Bruch & Schimp. – A, C, N

***Catagoniopsis berteroana*** (Mont.) Broth. – A, B, C, N

***Catagonium nitens*** (Brid.) Cardot – A, B, C, N

var. ***myurum*** (Cardot & Thér.) S.H.Lin – N

***Catagonium nitidum*** (Hook.f. & Wilson) Broth. – N

***Ceratodon purpureus*** (Hedw.) Brid. – A, B, C, N

subsp. ***convolutus*** (Reichardt) Burley – A, B, C, N

subsp. ***stenocarpus*** (Bruch & Schimp.) Dixon – A, B, C, N

***Chileobryon callicostelloides*** (Broth. ex Thér.) Enroth – A, B, C, N

***Chorisodontium aciphyllum*** (Hook.f. & Wilson) Broth. – N

***Chrysoblastella chilensis*** (Mont.) Reimers – A, B, C, N

***Cratoneuron filicinum*** (Hedw.) Spruce – B, N, A

***Cratoneuropsis relaxa*** subsp. **minor** (Wilson & Hook.f.) Ochyra – A, B, C, N

***Cryphaea consimilis*** Mont. – A, B, C, N

***Cryphaeophilum molle*** (Dusén) M.Fleisch. – A, B, C, N

***Cryptopapillaria penicillata*** (Dozy & Molk.) M.Menzel – A, C

*Daltonia gracilis Mitt. – A, B, N (considered a synonym of *Daltonia marginata* Griff. by P. Majestyk 2011).

***Daltonia marginata*** Griff. – **A (33520)** (det. Majestyk (2011) from MO specimen cited in publication).

**Daltonia ovalis Taylor – A, B (reported by He (1998) as *Daltonia trachydonta* Mitt. but unable to confirm specimens which were not cited).

***Daltonia splachnoides*** (Sm.) Hook. & Taylor – **A (33611)** (det. Majestyk (2011) from MO specimen cited in publication).

*Daltonia trachyodonta Mitt. – A, B (considered a synonym of *Daltonia ovalis* Taylor by Majestyk 2011).

***Dendrocryphaea cuspidata*** (Sull.) Broth. – A, B, C, N

+***Dendrocryphaea gorveana*** (Mont.) Paris & Schimp. – **C (32750** Coll. *Larraín*)

***Dendrocryphaea lechleri*** (M.Fleisch.) Paris & Schimp. ex Thér. – A, C

***Dendroligotrichum dendroides*** (Brid. ex Hedw.) Broth. – A, B

***Dicranella campylophylla*** (Taylor) A.Jaeger – A, B, N

*Dicranella harrisii (Müll.Hal.) Broth. – C (excluded from Chile—see Ireland et al. 2010, p. 44).

***Dicranella hookeri*** (Müll.Hal.) Cardot – **A (33219)**, B, C, **N (31068a, 36218)**

***Dicranella vaginata*** (Hook.) Cardot – A, B (Syn. *Anisothecium vaginatum* (Hook.) Mitt.).

***Dicranoloma billardierei*** (Brid.) Paris – A, C

***Dicranoloma chilense*** (De Not.) Ochyra & Matteri – A, B

***Dicranoloma dusenii*** (Broth.) Broth. – A

***Dicranoloma perremotifolium*** (Dusén) Broth. – A, B

var. ***fragile*** (Dusén) Thér. – A

***Didymodon australasiae*** (Hook. & Grev.) R.H.Zander – **A (33179**, C, **N (32536, 34720)**

***Didymodon deciduus*** R.H.Zander – B

***Didymodon fuscus*** (Müll.Hal.) J.A.Jiménez & Cano – **A (33156, 33159, 33673)**, B, C, N

***Didymodon rigidulus*** Hedw. – A, B, N

***Didymodon vinealis*** (Brid.) R.H.Zander – B, C

***Diphyscium pilmaiquen*** (Crosby) Magombo – A

***Distichophyllum krausei*** (Lorentz) Mitt. – N

***Ditrichum conicum*** (Mont.) Mitt. – **A (33114)**, B, **N (31556)**

***Ditrichum cylindricarpum*** (Müll.Hal.) F.Muell. – A, B, N

***Ditrichum difficile*** (Duby) M.Fleisch. – A, B, C, N

***Ditrichum hallei*** Cardot & Broth. – N

***Ditrichum hookeri*** (Müll.Hal.) Hampe – A, B, N

***Drepanocladus aduncus*** (Hedw.) Warnst. – B

***Drepanocladus polygamus*** (Schimp.) Hedenäs – C, N (Syn. *Campylium polygamum* (Schimp.) C.E.O.Jensen).

***Drummondia obtusifolia*** Müll.Hal. – B, N

***Dryptodon austrofunalis*** (Müll.Hal.) Ochyra & Żarnowiec – A, B, C, N (Syn. *Grimmia austrofunalis* Müll.Hal.).

***Dryptodon navicularis*** (Herzog) Ochyra & Żarnowiec – B (Syn. *Grimmia navicularis* Herzog).

***Dryptodon trichophyllus*** (Grev.) Brid. – A, B, C, N (Syn. *Grimmia trichophylla* Grev.).

***Encalypta ciliata*** Hedw. – B

***Entosthodon apophysatus*** (Taylor) Mitt. – B, C

***Entosthodon laevis*** (Mitt.) Fife – C

***Entosthodon obtusifolius*** Hook.f. – A, C, **N (32721, 34919)**

***Eriodon conostomus*** Mont. – A, B

***Eucamptodon perichaetialis*** (Mont.) Mont. – A, B, C, N

***Eucladium verticillatum*** (Brid.) Bruch & Schimp. – N

***Eurhynchiella acanthophylla*** (Mont.) M.Fleisch. – A, B, C, N

***Eurhynchium corralense*** (Lorentz) A.Jaeger – A, B, C, N

***Eurhynchium fuegianum*** Cardot – N

***Eustichia longirostris*** (Brid.) Brid. – A, B, C, N

***Fabronia ciliaris*** (Brid.) Brid. – B, C, N

var. ***wrightii*** (Sull.) W.R.Buck – B

***Fabronia jamesonii*** Taylor – A, B, C, N

***Fissidens asplenioides*** Hedw. – A, B, C, N

***Fissidens bryoides*** var. ***pusillus*** (Wilson) Pursell – A, **B (34818, 34857, 34944)**, C, **N (30614, 30647)**

var. ***viridulus*** (Sw.) Broth. – A, **B (34814, 34884, 34954, 34983, 35053, 35072, 35229, 35522, 35697, 35704, 35742, 35769)** C, **N (34600, 35798, 35799, 35969)**

***Fissidens crispus*** Mont. – A, B, C, **N (32301)**

***Fissidens curvatus*** Hornsch. – A, B, C, N

***Fissidens maschalanthus*** Mont. – A

***Fissidens oblongifolius*** Hook.f. & Wilson – A, B, C, N

***Fissidens rigidulus*** Hook.f. & Wilson – A, B, C, N

***Fissidens scalaris*** Mitt. – B, C

***Fissidens serratus*** Müll.Hal. – C

var. ***leptochaete*** (Dusén) Brugg.-Nann. & Pursell – B, C

***Fissidens taxifolius*** Hedw. – C

***Funaria chilensis*** (Thér.) Thér. – C, N

*Funaria commixta Thér. – C (identification of this species by Ireland & Bellolio *31631* was reidentified as *Funaria costesii* Thér. according to Cuvertino et. al. 2012).

***Funaria costesii*** Thér. – C

***Funaria hygrometrica*** Hedw. – A, B, C, N

***Gemmabryum dichotomum*** (Hedw.) J.R.Spence & H.R.Ramsay – C (Syn. *Bryum dichotomum* Hedw.).

***Gemmabryum valparaisense*** (Thér.) J.R.Spence – C (Syn. *Bryum valparaisense* Thér.).

***Glyphothecium gracile*** (Hampe) Broth. – A, B, C, N

***Grimmia laevigata*** (Brid.) Brid. – B, N

***Grimmia reflexidens*** Müll.Hal. – B, N

*Gymnostomum aeruginosum Sm. – B, C, N (excluded from South America by Cano & Jiménez 2013).

***Gymnostomum calcareum*** Nees & Hornsch. – **A (33193)**, B, C, N

*Gymnostomum tenerrimum (Müll.Hal.) Wijk & Margad. – C, N (considered a synonym of *Gymnostomum calcareum* by Cano and Jiménez 2013).

***Haplohymenium longinerve*** (Broth.) Broth. – B

***Hebantia rigida*** (Lorentz) G.L.Merr. – A, C, N

***Hedwigidium integrifolium*** (P.Beauv.) Dixon – B, N

***Hennediella arenae*** (Besch.) R.H.Zander – **A (31261, 33555, 33651, 33670)**, C, N (Syn. *Tortula polycarpa* Dusén).

***Hennediella kunzeana*** (Müll.Hal.) R.H.Zander – A, B, C, N

***Holodontium strictum*** (Hook.f. & Wilson) Ochyra – N

***Hymenodontopsis mnioides*** (Hook.) N.E.Bell, A.E.Newton & D.Quandt – A, B, C, N (Syn. *Pyrrhobryum mnioides* (Hook.) Manuel).

***Hypnodendron microstictum*** Mitt. ex A.Jaeger & Sauerb. – A, B, C, N

***Hypnum cupressiforme*** Hedw. – A, B, C, N

var. ***filiforme*** Brid. – A, B, N

***Hypopterygium didictyon*** Müll.Hal. – B, C

***Imbribryum clavatum*** (Schimp.) J.R.Spence & H.P.Ramsey – **B (35236)**, C, **N (34743)** (Syn. *Bryum clavatum* (Schimp.) Müll.Hal.).

***Imbribryum laevigatum*** (Hook.f. & Wilson) J.R.Spence & H.P.Ramsey – A, B (Syn. *Bryum laevigatum* Hook.f. & Wilson).

***Isopterygiopsis pulchella*** (Hedw.) Z.Iwats. – B

***Juratzkaea seminervis*** (Kunze ex Schwägr.) Lorentz – A, B, C, N

***Kindbergia praelonga*** (Hedw.) Ochyra – A, B, C

***Leptobryum pyriforme*** (Hedw.) Wilson – N

***Leptodictyum riparium*** (Hedw.) Warnst. – B, N

***Leptodon smithii*** (Hedw.) F.Weber & D.Mohr – A, B, C, N

***Leptodontium proliferum*** Herzog – A

***Leptostomum menziesii*** R.Br. – A, B, N

***Leptostomum splachnoideum*** Hook. & Arn. – A, C, N

***Leptotheca gaudichaudii*** Schwägr. – A, B, C, N

***Lepyrodon hexastichus*** (Mont.) Wijk & Margad. – A, B, C, N

***Lepyrodon lagurus*** (Hook.) Mitt. – A, B, N

***Lepyrodon parvulus*** Mitt. – A, B, C, N

***Lepyrodon patagonicus*** (Cardot & Broth.) B.H.Allen – A, B, C

***Lepyrodon tomentosus*** (Hook.) Mitt. – A, N

***Looseria orbiculata*** (Thér.) D.Quandt, Huttunen, Tangney & M.Stech – A, B, C, N (Syn. *Lembophyllum orbiculatum* (Thér.) Tangney).

***Lopidium concinnum*** (Hook.) Wilson – A, B

***Macrocoma sullivantii*** (Müll.Hal.) Grout – A, B, C, N

***Macromitrium crassiusculum*** Lorentz – A, B, C, **N (34703)**

***Macromitrium krausei*** Lorentz – N

***Macromitrium microcarpum*** Müll.Hal. – A, C, N

***Microcampylopus leucogaster*** (Müll.Hal.) B.H.Allen – A, C, N

***Neckera chilensis*** Schimp. ex Mont. – A, B, C, N

***Neckera scabridens*** Müll.Hal. – A, B, C, N

***Notoligotrichum minimum*** (Cardot) G.L.Sm. – B

***Oedipodium griffithianum*** (Dicks.) Schwägr. – B

***Oligotrichum canaliculatum*** (Hook. & Arn.) Mitt. – A, B, C, N

***Orthodontium gracile*** (Wilson) Schwägr. ex B.S.G. – B, C, N

*Orthodontium pellucens (Hook.) B.S.G. – A, B, C, N (reported by Ireland et al. 2006 from all 4 provinces but all collections are *Eucamptodon perichaetialis*, which were misidentified).

*Orthotrichum aequaetoreum Mitt. – N (reported by Ireland et al. 2006, but misidentified specimen 30686 is *Orthotrichum freyanum* and misreported specimen 30687 is *Orthotrichum latimarginatum*).

***Orthotrichum anaglyptodon*** Cardot & Broth. – B, N

***Orthotrichum araucarieti*** Müll.Hal. – B, N

***Orthotrichum assimile*** Müll.Hal. – A, B, C, N

***Orthotrichum brotheri*** Dusén ex Lewinsky – A, B, C, N

***Orthotrichum densum*** Lewinsky – N

***Orthotrichum elegantulum*** Schimp. ex Mitt. – B, **C (32891)**, N

***Orthotrichum freyanum*** Goffinet, W.R.Buck & M.A.Wall – B, **N (30686)**

***Orthotrichum hortense*** Bosw. – B

***Orthotrichum incanum*** Müll.Hal. – B, C, N

***Orthotrichum latimarginatum*** Lewinsky – N

***Orthotrichum laxifolium*** Wilson – N

***Orthotrichum ludificans*** Lewinsky – B

***Orthotrichum*** cf. ***pariatum*** Mitt. – B, N

***Orthotrichum perexiguum*** Dusén ex Lewinsky – B

***Orthotrichum rupestre*** Schleich. ex Schwägr. – B, N

***Orthotrichum tristriatum*** Lewinsky – A, B, C, N

***Orthotrichum truncatum*** Lewinsky & Deguchi – A, B, C

***Papillaria flexicaulis*** (Wilson) A.Jaeger – B, C (Syn. *Meteorium flexicaule* Wilson).

***Philonotis elongata*** (Dism.) H.A.Crum & Steere – C

***Philonotis esquelensis*** Matteri – C (reported by Jimenez et al. 2014)

***Philonotis krausei*** (Müll.Hal.) Broth. – A, B, C, N

***Philonotis nigroflava*** Müll.Hal. – A, B, N

***Philonotis scabrifolia*** (Hook.f. & Wilson) Braithw. – A, B, C, N

***Philonotis vagans*** (Hook. & Wilson) Mitt. – A, B, N

***Physcomitrium badium*** Broth. – C

***Physcomitrium lorentzii*** Müll.Hal. – A

***Plagiothecium denticulatum*** (Hedw.) Schimp. – N

***Plagiothecium orthocarpum*** Mitt. – C

***Plagiothecium ovalifolium*** Cardot – B

***Platyneuron praealtum*** (Mitt.) Ochyra & Bedn.-Ochyra – A, B, N

*Pleuridium andinum Herzog – C, N (excluded from Chile—see Ireland et al. 2010, p. 44, both collections, 31527 & 31965, are *Pleuridium subnervosum*).

***Pleuridium robinsonii*** (Mont.) Mitt. – A, B, C, N

***Pleuridium subnervosum*** (Müll.Hal.) A.Jaeger ex Paris – C, N (Syn. *Pleuridium macrothecium* Dusén).

***Pogonatum perichaetiale*** subsp. ***oligodus*** (Kunze ex Müll.Hal.) Hyvönen – B, C, N (Syn. *Pogonatum oligodus* (Kunze ex Müll.Hal.) Mitt.).

***Pohlia chilensis*** (Mont.) A.J.Shaw – A, B, C, N

***Pohlia cruda*** (Hedw.) Lindb. – A, B, C, N

***Pohlia nutans*** (Hedw.) Lindb. – A, N

***Pohlia wahlenbergii*** (F.Weber & D.Mohr) A.L.Andrews – A, B, C, N

+***Pohlia wilsonii*** (Mitt.) Ochyra – **C (30408, 31025, 31689, 31761, 32081, 32219)**, **N (32519, 32778)**

***Polytrichastrum alpinum*** (Hedw.) G.L.Sm. – N

***Polytrichum juniperinum*** Hedw. – A, B, C, N

***Polytrichum piliferum*** Scherb. ex Hedw. – A, B, N

***Polytrichum strictum*** Menzies ex Brid. – N

***Porothamnium arbusculans*** (Müll.Hal.) M.Fleisch. – A, B, C, N

***Porothamnium leucocaulon*** (Müll.Hal.) M.Fleisch. – N

***Porothamnium panduraefolium*** (Müll.Hal.) M.Fleisch. – A, C, N

***Porothamnium valdiviae*** (Müll.Hal.) M.Fleisch. – B, **C (31959)**, N

***Porotrichum chilense*** Thér. – B, N

***Porotrichum korthalsianum*** (Dozy & Molk.) Mitt. – A, B, C

***Porotrichum lancifrons*** (Hampe) Mitt. – A, B, C, N

***Pseudocrossidium crinitum*** (Schultz) R.H.Zander – B, C, N

***Pseudocrossidium santiagensis*** (Broth.) M.J.Cano – C

***Pseudotaxiphyllum elegans*** (Brid.) Z.Iwats. – A

***Ptychomitrium deltorii*** (Thér.) Broth. – C

***Ptychomitrium sellowianum*** (Müll.Hal.) A.Jaeger – A, C, N

***Ptychomnion cygnisetum*** (Müll.Hal.) Kindb. – A

++***Ptychostomum bimum*** (Schreber) J.R.Spence – **C (33934)**

++***Ptychostomum creberrimum*** (Taylor) J.R.Spence & H.P.Ramsey – **B (35465)**

++***Ptychostomum inclinatum*** (Sw. ex Brid.) J.R.Spence – **N (30585, 30617)**

***Ptychostomum orthothecium*** (Cardot & Broth.) Holyoak & N.Pederson – C (Syn. *Bryum orthothecium* Cardot & Broth.).

***Ptychostomum turbinatum*** (Hedw.) J.R.Spence – N (Syn. *Bryum turbinatum* (Hedw.) Turner).

*Racomitrium crispipilum (Taylor) A.Jaeger – A, B, N (excluded from Chile flora—see Larraín 2012).

*Racomitrium crispulum (Hook.f. & Wilson) Wilson – N (excluded from Chile flora—see Ireland et al. 2010, p. 43).

***Racomitrium didymum*** (Mont.) Lorentz – A, B, N (Syn. *Bucklandiella didyma* (Mont.) Bedn.-Ochyra & Ochyra).

***Racomitrium geronticum*** Müll.Hal. – A, B, C, N (Syn. Racomitrium lanuginosum subsp. geronticum (Müll.Hal.) Vitt & C.Marsh; Syn. *Racomitrium patagonicum* Bedn.-Ochyra & Ochyra).

*Racomitrium lanuginosum (Hedw.) Brid. – B (excluded from Chile flora—see Larraín 2012).

***Racomitrium lamprocarpum*** (Müll.Hal.) A.Jaeger – A, B, N (Syn. *Bucklandiella lamprocarpa* (Müll.Hal.) Bedn.-Ochyra & Ochyra).

***Racomitrium orthotrichaceum*** (Müll.Hal.) Paris – N (Syn. *Bucklandiella orthotrichacea* (Müll.Hal.) Bedn.-Ochyra & Ochyra).

***Racomitrium rupestre*** (Hook.f. & Wilson) Hook.f. & Wilson – B, N (Syn. *Bucklandiella rupestris* (Hook.f. & Wilson) Bedn.-Ochyra & Ochyra).

***Racomitrium subcrispipilum*** Müll.Hal. – B, N (Syn. *Racomitrium striatipilum* Cardot).

***Racopilum cuspidigerum*** (Schwägr.) Ångström – A, C

***Renauldia chilensis*** Thér. – C

***Rhabdoweisia crispata*** (Dicks.) Lindb. – C

***Rhabdoweisia fugax*** (Hedw.) Bruch & Schimp. – A, C

***Rhaphidorrhynchium amoenum*** (Hedw.) M.Fleisch. – A, C

***Rhaphidorrhynchium leptophyllum*** (Mitt.) Broth. – C

***Rhaphidorrhynchium callidum*** (Mont.) Broth. – A, B, C, N

***Rhaphidorrhynchium scorpiurus*** (Mont.) Broth. – A (Syn. *Sematophyllum scorpiurus* (Mont.) Mitt.).

***Rhynchostegium complanum*** (Mitt.) A.Jaeger – C

***Rigodium adpressum*** Zomlefer – A, B, C, N

***Rigodium brachypodium*** (Müll.Hal.) Paris – A, B, C, N

***Rigodium implexum*** Kunze ex Schwägr. – A, C, N

***Rigodium pseudothuidium*** Dusén – C, N

***Rigodium tamarix*** Müll.Hal. – A, B, C, N

***Rigodium toxarion*** (Schwägr.) A.Jaeger – A, B, C, N

***Rosulabryum billardieri*** (Schwägr.) J.R.Spence – A, B, C, N (Syn. *Bryum billardieri* Schwägr.).

+***Rosulabryum campylothecium*** (Taylor) J.R.Spence – **C (31983)**, **N (31550)** (Syn. *Bryum campylothecium* Taylor).

***Rosulabryum capillare*** (Hedw.) J.R.Spence – **B (34950)**, C (Syn. *Bryum capillare* Hedw.).

+***Rosulabryum torquescens*** (Bruch & Schimp.) J.R.Spence – **C (32819)** (Syn. *Bryum torquescens* Bruch & Schimp.).

+***Rosulabryum viridescens*** (Welw. & Duby) Ochyra – **N (35912)** (Syn. *Bryum viridescens* Welw. & Duby)

***Sanionia symmetrica*** (Renauld & Cardot) Wheldon – N

***Sanionia uncinata*** (Hedw.) Loeske – B, N

***Schimperobryum splendidissimum*** (Mont.) Margad. – A, B, C, N

var. ***perdentatum*** Matteri – C

***Schistidium apocarpum*** (Hedw.) Bruch & Schimp. – B, C, N

***Schistidium rivulare*** (Brid.) Podp. – B, C, N

***Schistidium falcatum*** (Hook.f. & Wilson) B.Bremer – B, N

***Schistidium scabripes*** (E.B.Bartram) Deguchi – N

***Schizymenium multiflorum*** (E.B.Bartram) A.J.Shaw – N

***Sciuro plumosum*** (Hedw.) Ignatov & Huttunen – N

***Scouleria patagonica*** (Mitt.) A.Jaeger – B, N

+++***Sematophyllum harpidioides*** (Renauld & Cardot) F.D.Bowers – **A (33095, 33109, 33464, 33465, 33513, 33552, 33596, 33640, 33648)**

***Sphagnum fimbriatum*** Wilson – A, N (as *Sphagnum flexuosum* Dozy & Molk.—see Ireland et al. 2010, p. 43).

*Sphagnum flexuosum Dozy & Molk. – N (excluded from Chile— see *Sphagnum fimbriatum* above).

***Sphagnum magellanicum*** Brid. – A

***Sphagnum recurvum*** var. ***brevifolium*** (Lindb.) Warnst. – A (reported by Ireland et al. (2006). Dick Andrus observed specimens at CONC (33090, 33091, 33093) and concluded they belong to an undescribed taxon in section Cuspidata).

***Sphagnum*** cf. ***subsecundum*** Nees – A

***Splachnobryum obtusum*** (Brid.) Müll.Hal. – C

***Symblepharis krausei*** (Lorentz) Ochyra & Matteri – A, B, N (Syn. *Symblepharis luteovirens* (E.B.Bartram) Ochyra & Matteri; Syn. *Oncophorus luteovirens* E.B.Bartram).

***Syntrichia breviseta*** (Mont.) M.J.Cano & M.T.Gallego – **A (31213, 33022, 33029, 33402, 33450, 33574, 33689)**, B, **C (30896, 32880, 34010, 34035), N (32625, 32637, 32658, 32693)**

+***Syntrichia costesii*** (Thér.) R.H.Zander – **N (35838, 35842, 35893)**

***Syntrichia epilosa*** (Broth. ex Dusén) R.H.Zander – A, **B (34842)**, C, N

***Syntrichia fragilis*** (Taylor) Ochyra – **A (33661, 33676)**, B, C, N

***Syntrichia glacialis*** (Kunze ex Müll.Hal.) R.H.Zander – A, B, C, N

***Syntrichia laevipila*** Brid. – A, **B (32048, 35020, 35204)**, C, N

***Syntrichia papillosa*** (Wilson) Jur. – A, C

***Syntrichia princeps*** (De Not.) Mitt. – B

***Syntrichia pseudorobusta*** (Dusén) R.H.Zander – A, B, C, N

***Syntrichia robusta*** (Hook. & Grev.) R.H.Zander – A, B, C, N

var. ***recurva*** (Lightowlers) R.H.Zander – C, N

***Syntrichia ruralis*** (Hedw.) F.Weber & D.Mohr – A, B, N

***Syntrichia scabrella*** (Dusén) R.H.Zander – **A (33181, 33784), B (34293, 35202)**, C, N

***Syntrichia scabrinervis*** (Müll.Hal.) R.H.Zander – A, B, **C (32092, 32112, 32182), N (32437, 32552, 32689, 34639, 35774)**

***Syntrichia serrulata*** (Hook. & Grev.) M.J.Cano – A, B, C, N

+***Syntrichia socialis*** (Dusén) R.H.Zander – **B (35954)**

***Syntrichia squarripila*** (Thér.) Herzog – **A (32963, 33247, 33575, 33679)**, B, **C (30941, 31464, 31931, 31936, 32082, 32133, 32143, 32158, 32159, 32164, 32181, 32256, 32256a, 32258, 32274, 32280, 32881, 33855, 34009, 34032, 34041, 34042)**, N

***Thamniopsis incurva*** (Hornsch.) W.R.Buck – C

***Thamnobryum fasciculatum*** (Sw. ex Hedw.) I.Sastre – A, B, C, N

***Thuidiopsis dusenii*** (Broth.) Broth. – A

***Thuidiopsis furfurosa*** (Hook.f. & Wilson) M.Fleisch. – A

***Thuidiopsis sparsa*** (Hook.f. & Wilson) Broth. – A, B, N

***Tortella tortuosa*** (Hedw.) Limpr. – B

***Tortula jaffuelii*** Thér. – A, C, N

***Tortula muralis*** Hedw. – A, B, C, N

***Tortula platyphylla*** Mitt. – C

***Tortula truncata*** (Hedw.) Mitt. – B, C

***Trichostomum elliottii*** Broth. ex Dusén – C

***Triquetrella patagonica*** Müll.Hal. – A, B, C, N

fo. ***filicaulis*** (Dusén) Herzog – C

***Ulota macrodontia*** Dixon & Malta – A, N

***Ulota rufula*** (Mitt.) A.Jaeger – A, B, N

***Vittia pachyloma*** (Mont.) Ochyra – A, B, C, N

***Warnstorfia exannulata*** (Schimp.) Loeske – A

***Weissia controversa*** Hedw. – A, B, C, N

***Weymouthia cochlearifolia*** (Schwägr.) Dixon – A

***Weymouthia mollis*** (Hedw.) Broth. – A

***Willia brachychaete*** (Dusén) R.H.Zander – **A (32960, 33453, 33728)**, B, N

***Zygodon hookeri*** var. ***leptobolax*** (Müll.Hal.) Calabrese – A, B, C, N

***Zygodon obtusifolius*** Hook. – A (Syn. *Bryomaltaea obtusifolia* (Hook.) Goffinet)

***Zygodon papillatus*** Mont. – A, B, C, N

***Zygodon pentastichus*** (Mont.) Müll.Hal. – A, B, C, N

### Biogeographical analyses of the Bío-Bío mosses

**Figure 2.**
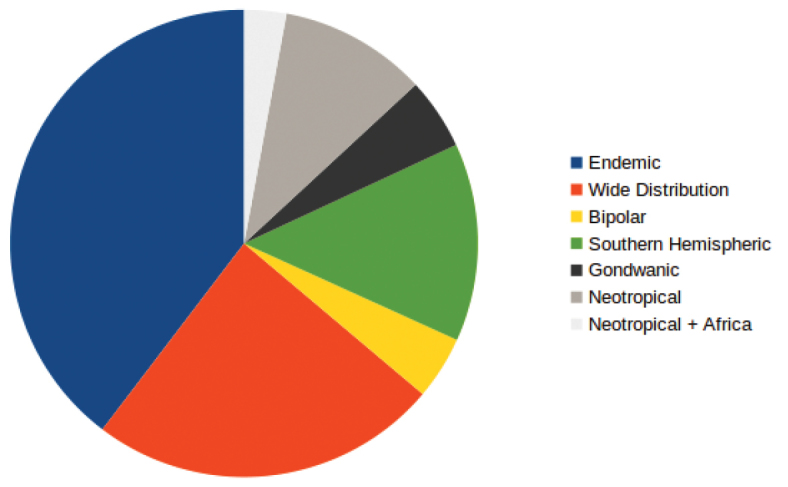
Proportion of the biogeographic components in the total moss flora of Bío-Bío Region.

From the biogeographical analyses of the taxa found in the Bío-Bío Region, 39.65% of the taxa are endemic to southern South America and adjacent areas, 24.19% of the taxa are bipolar, 13.7% are Southern Hemispheric taxa, 10.2% correspond to Neotropical taxa, 4.95% are Gondwanic taxa, and only 2.91% of the taxa are shared between southern South America, the Neotropics and Africa (Fig. 2).

In terms of the distribution of mosses within the Bío-Bío Region, we have seen that the most diverse province is Ñuble with 226 taxa, followed by Bío-Bío Province (210), Concepción Province (200), and Arauco Province (197). These numbers may change with the more collecting still needed in all the four provinces.

## Discussion

After the examination of more than 6,000 collections from Bío-Bío Region, a total of 343 moss taxa are reported in this paper. This number represents a major increase from the 190 taxa reported by He (1998) in the last checklist done before the beginning of this project. Several taxa had to be reevaluated, several names had to be changed and updated due to recent revisionary studies made by colleagues worldwide, and some taxa previously reported for the region had to be excluded from the Bío-Bío moss flora. A number of our colleagues identified difficult groups, like the Pottiaceae (María Jesús Cano, Mayte Gallego, Juan Jiménez, Richard Zander), *Fissidens* (Ron Pursell †), Bryaceae (John Spence), Dicranaceae (J.-P. Frahm †), and *Bartramia*, *Sematophyllum* (Bruce Allen).

As it has been noted elsewhere (Seki 1974, Villagrán et al. 2003, Larraín 2005, 2016), the dominating biogeographical component in any central or southern Chile region corresponds to the endemic element, reaching almost the 40% of taxa for the mosses of the Bío-Bío region. The second most represented element is the widely distributed species, that includes several recently introduced taxa or species mostly associated with human disturbances. As it happens in other regions of Chile with the mosses, it is interesting that the Southern Hemisphere plus the Gondwanic elements sum up almost twice the number of taxa that shows a southern South America-Neotropical distribution. Finally, there is a small number of species (n=10) shared between southern South America and the African continent.

Three taxa are still troublesome for us but they have been included in the list. One of these is *Andreaea rupestris*, a species that has been excluded from the Southern Hemisphere by both Vitt (1980) and Murray (2006). The latter authors suggest that the records reported as *Andreaea rupestris* from the Southern Hemisphere might correspond to *Andreaea mutabilis* Hook.f. & Wilson or some other taxon with ecostate leaves. Another doubtful taxon is *Plagiothecium denticulatum*, reported for the first time for Chile by Mitten (1869), as *Plagiothecium donnianum* (Sm.) Mitt. from Cape Horn, and subsequently reported several times by other authors (Müller 2009a). This taxon is not even mentioned in the monograph of Plagiotheciaceae for the Flora Neotropica project (Buck and Ireland 1989), and presently it is considered to be a boreal taxon. The third problematic taxon is *Thamniopsis incurva*, a widespread Neotropical species whose type was apparently collected in Chile by Chamisso (judging from the label of the type), but it has never been found again in Chile. It is possible that the original specimen of Chamisso was mislabeled and collected somewhere else along the South American coast.
